# Characterization of SLC6A1 mutations associated with schizophrenia using SH-SY5Y-based cell lines Ekaterina Marilovtseva1

**DOI:** 10.1192/j.eurpsy.2025.1532

**Published:** 2025-08-26

**Authors:** E. Marilovtseva, D. Abashkin, V. Golimbet

**Affiliations:** 1 Clinical Genetics Laboratory, Mental Health Research Center, Moscow, Russian Federation

## Abstract

**Introduction:**

SLC6A1 is a GABA transporter which, being expressed in neurons and glial cells, removes GABA from extracellular space, preventing the spread of the inhibitory transmission within the brain. Recently, A93T, R211C, and W495L *de novo* mutations in SLC6A1 have been demonstrated to be involved in schizophrenia pathogenesis. However, the mechanism of action of SLC6A1 in schizophrenia is still to be determined.

**Objectives:**

To establish a series of SH-SY5Y-based cell lines expressing wt *SLC6A1* and its forms with single A93T, R211C or W495L mutations and their combinations, and to further characterize the properties and the subcellular localization of the resulting proteins.

**Methods:**

The chosen variants of *SLC6A1* tagged with N-terminal FLAG were cloned into a lentiviral plasmid under the TRE promoter and used for transfection of HEK293T with further SH-SY5Y transduction. *SLC6A1* expression was initiated by adding 20 μg/mL of doxycycline to the cells. After 48 hours of treatment the cells were either used for qPCR and Western blot analysis, or stained.

**Results:**

All cell lines expressed *SLC6A1* forms efficiently. SLC6A1 with single mutations, SLC6A1^R211C;W495L^, and SLC6A1^A93T; R211C;W495L^ were present by the same ≈67kDa form as wt SLC6A1, while SLC6A1^A93T;R211C^ resulted in an additional band at ≈130kDa, indicating either the presence of PTM, or the formation of a homodimer. SLC6A1^A93T;W495L^ gave no protein product, probably, due to its proteolytic degradation. Interestingly, wt SLC6A1, SLC6A1^R211C^, and SLC6A1^A93T;R211C^ were detected in neurites, while SLC6A1^A93T^, SLC6A1^W495L^, SLC6A1^R211C;W495L^, and SLC6A1^A93T;R211C;W495L^ were mainly found in cytoplasm, which might indicate that these mutations might affect the function of the protein.

**Conclusions:**

Having established a series of SH-SY5Y-based cell lines expressing SLC6A1 with schizophrenia-associated mutations, we demonstrated the effect of the latter on the protein’s subcellular localization. Based on our observations, we speculate that R211C has the mildest effect on the localization and function of SLC6A1 and can even partially compensate that of A93T, but not W495L. We also suggest that SLC6A1 with the A93T;W495L combination undergoes proteolytic degradation, most likely, due to the defects in its structure.

**Disclosure of Interest:**

None Declared

